# Liver Cholestasis Secondary to Syphilis in an Immunocompetent Patient

**DOI:** 10.1155/2018/8645068

**Published:** 2018-10-22

**Authors:** Nazneen Hussain, Samuel O. Igbinedion, Richie Diaz, J. S. Alexander, Moheb Boktor, Kurt Knowles

**Affiliations:** ^1^Department of Gastroenterology and Hepatology, Louisiana State University Health Sciences Center, Shreveport, LA, USA; ^2^Department of Internal Medicine, Louisiana State University Health Sciences Center, Shreveport, LA, USA; ^3^School of Medicine, Louisiana State University Health Sciences Center, Shreveport, LA, USA; ^4^Department of Molecular and Cellular Physiology, Louisiana State University Health Sciences Center, Shreveport, LA, USA; ^5^Department of Pathology, Louisiana State University Health Sciences Center, Shreveport, LA, USA

## Abstract

Liver involvement is a known feature of secondary syphilis. The prevalence of hepatitis in secondary syphilis ranges broadly from 1 to 50%. We report a case of a 37-year-old man with type 1 diabetes mellitus and sickle cell trait presenting with jaundice and acute liver cholestasis. Abdominal ultrasound revealed mild hepatic fatty infiltration. RPR and Treponema pallidum IgG results were positive with a reflex titer of 1:64. Liver biopsy revealed chronic hepatitis with normal hepatic architecture, Kupffer cell hyperplasia, hepatic cholestasis, and ductal proliferation suggestive of syphilitic hepatitis.

## 1. Introduction

Causes of hepatic dysfunction deemed as hepatitis include but are not limited to metabolic causes, drugs or toxins, alcohol, malignancy, ischemia, infection, and genetic causes [1]. Serum liver biochemical tests, also known as “liver function tests,” are very useful in the evaluation of patients who present with hepatic dysfunction [2]. The usefulness of these tests is to help identify liver disease, discern the cause of the dysfunction, and monitor for worsening progression or response after therapy [2]. The liver could be affected by primary infection, extrahepatic infection or systemic effects of infection. These different modalities of infections could lead to hepatic dysfunction [1]. The severity could range from mild derangements in the serum biochemical tests to jaundice, and in rare cases, acute liver failure [1]. Abnormalities present for more than six months are deemed chronic [2].

Secondary syphilis usually manifests as a systemic stage of the syphilitic infection [3]. Greater than 95% of cases of secondary syphilis manifest in the skin and mucous membranes [3]. Syphilitic hepatitis, characterized by a high serum alkaline phosphatase, only occurs in up to 50% of the cases [3]. Due to a wide array of potential causes, determining the etiology of hepatic dysfunction can be a diagnostic challenge for the clinician. Furthermore, identifying syphilitic hepatitis as a cause of hepatic dysfunction is even more challenging if there are no accompanying systemic signs of syphilitic infection. We report a fascinating case of liver cholestasis secondary to syphilis in an immunocompetent patient.

## 2. Case Report

A 37-year-old African American man with a history of type 1 diabetes and sickle cell trait was referred to the Gastroenterology service for ERCP/EUS to evaluate jaundice. He presented with right upper quadrant (RUQ) abdominal pain with associated nausea and vomiting ongoing in the past ten days. He denied the use of tobacco, alcohol, or other illicit drugs. The patient reported several female sexual partners in the past six months. Physical exam revealed scleral icterus and RUQ abdominal tenderness. Laboratory studies were notable for alanine aminotransferase (ALT) of 59 U/L, aspartate aminotransferase (AST) of 39 U/L, total bilirubin of 11.4 mg/dL, and alkaline phosphatase (ALP) of 657 U/L. His hepatitis A antibody, hepatitis B surface antigen, hepatitis B surface antibody, and hepatitis C antibody were negative. HIV-1 and HIV-2 antibodies were negative. Serum autoimmune markers, alpha-1 antitrypsin, iron profile, and ceruloplasmin were also negative. Antimitochondrial antibody was positive and smooth muscle antibody was weakly positive. Lactate dehydrogenase and haptoglobin levels were within normal limits.

**Figure 1:**
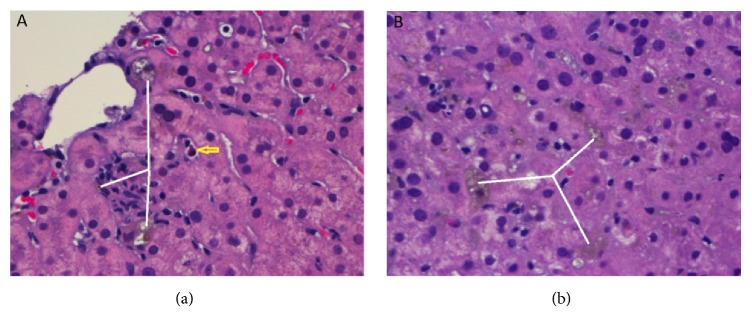
Liver core biopsy demonstrated (a) cholestasis and inflammatory infiltrates and (b) moderate canalicular cholestasis. The white lines indicate cholestasis and bile plugging. The yellow arrow indicates an eosinophil. H&E stain high power (400x) highlighted these findings.

Liver ultrasound showed mild hepatic fatty infiltration without biliary obstruction or stones. Magnetic resonance cholangiopancreatography (MRCP) was negative for biliary or pancreatic ductal dilation. RPR returned positive with a reflex titer of 1:64. Treponema pallidum IgG was sent for confirmation and it was reactive. Liver biopsy demonstrated chronic hepatitis with normal hepatic architecture, Kupffer cell hyperplasia, hepatic cholestasis and ductal proliferation (Figure 1). Iron stain was positive. Periodic acid-Schiff and Periodic acid-Schiff-diastase stains were negative for alpha 1 anti-trypsin granules. Warthin starry stain was negative. Immunochemical stain for* Treponema pallidum *revealed no organisms. These findings were suggestive of syphilitic hepatitis. Patient had a reported allergy of pruritus to penicillins in the past. The allergist was consulted and patient underwent a challenge with oral penicillin and tolerated it well, no reaction was noted. The patient was given a single dose treatment: penicillin G 2.4 million units intramuscular route (IM) once. He showed symptomatic improvement subsequently, including resolution of nausea/RUQ abdominal pain. He was discharged to be followed up outpatient. Follow-up visit in two weeks revealed improvement in serum biochemical tests with ALT of 45 U/L, AST of 39 U/L, ALP of 298 U/L, total bilirubin 1.7 mg/dL, and absence of abdominal pain.

## 3. Discussion

The work-up of elevated liver serum biochemical tests begins with the initial history and physical exam. Alcohol abuse which is a very common cause of liver disease can be elicited by the acknowledgement of significant alcohol consumption [4]. Exposure to hepatotoxic drugs and risk factors for viral hepatitis which include parenteral drug use, travel to regions endemic for hepatitis need to be identified [1]. The pattern of the liver tests abnormalities could suggest the etiology of the liver disease, classifying if it is hepatocellular, cholestatic, or an isolated hyperbilirubinemia [5]. The magnitude of the elevations and the pattern of other lab abnormalities guide the work-up of liver disease. Imaging can also provide additional benefit as well as a liver biopsy in patients who have had unrevealing work-up [6].

Our patient's history and physical, laboratory and pathologic findings highlight syphilitic hepatitis as the most likely cause of his liver cholestasis. Liver involvement is a known feature of secondary syphilis. However, this is only seen in 1 – 50% of patients with secondary syphilis [3]. The rash is the more common finding in patients with secondary syphilis [7]. The rash usually presents in any form; macular, annular, vesicular, pustular, papular, papulosquamous, or combinations of these [8]. Vesicular rashes are not very commonly seen [8]. Mullick et al. described the cases of seven HIV infected patients who presented with syphilitic hepatitis at George Washington University Medical Center during the time period 2001-2003 [9]. All of these patients described presented with a rash consistent with secondary syphilis on evaluation [9]. Interestingly, our patient had no significant mucocutaneous involvement on presentation to the hospital. It is, however, possible that the patient had lesions that he did not appreciate or that had resolved and thus were not identified. Given this rare presentation of secondary syphilis, making the diagnosis of syphilitic hepatitis, was quite challenging.

Syphilitic hepatitis has been defined in previous studies as a case of confirmed primary or secondary syphilis with a positive RPR and* T. pallidum *antibody, presence of a clinical illness consistent with syphilis, elevated liver tests including an alkaline phosphatase level which returns to normal following therapy, and exclusion of other causes of hepatitis [9, 10]. Our patient's presentation met these key features seen in syphilitic hepatitis. The patient presented with abnormal liver biochemical tests, especially the elevated alkaline phosphatase level. The pericholangiolar inflammation may cause a cholestatic picture and thus could explain this significant elevation in alkaline phosphatase [10]. Some of the diagnostic features of syphilitic hepatitis are obtained from the histologic examination of the liver. This shows focal necrosis in the periportal and centrilobular regions [1]. Inflammatory infiltrate including neutrophils, lymphocytes and eosinophils are typically seen [1]. Kupffer cell hyperplasia could also be observed. Figure 1 highlights some of these diagnostic features. Immunohistochemical stain only identifies spirochetes in about 50% of patients and, thus, has poor sensitivity for diagnosing syphilitic hepatitis [2].

This case is unique as we highlight syphilitic hepatitis in an immunocompetent patient who presented with jaundice. Our patient presented with an initial history and physical significant for the absence of constitutional symptoms or defining rash consistent with a sexually transmitted disease. Baveja et al. reported a case of syphilitic hepatitis presenting as the initial manifestation of secondary syphilis [11]. However the patient reported did present with a maculopapular rash. To our knowledge, this is the only case of syphilitic hepatitis presenting as the sole identifiable manifestation of secondary syphilis. Our patient had a rapid improvement in clinical and laboratory findings after penicillin G treatment. This clinical presentation should alert gastroenterologists to consider syphilis in the work-up of hepatitis even in patients without other manifestations of the disease.
